# Orchestrating Tumor Metastasis: Exosomes as Master Regulators of the Local and Distant Microenvironment

**DOI:** 10.7150/ijbs.128203

**Published:** 2026-02-18

**Authors:** Yuanke Zhao, Aiyu Liu, Xi Zhang, Anqi Zeng, Linjiang Song

**Affiliations:** 1 School of Medical and Life Sciences, Chengdu University of Traditional Chinese Medicine, Chengdu, China.; 2 Translational Chinese Medicine Key Laboratory of Sichuan Province, Sichuan Academy of Chinese Medicine Sciences, Sichuan Institute for Translational Chinese Medicine, Chengdu, Sichuan 610041, China.

**Keywords:** tumor microenvironment, cancer metastasis, exosomes, cancer therapy

## Abstract

Metastasis remains a critical challenge in oncology and constitutes the leading cause of cancer mortality. Recent studies have revealed that exosomes are involved in every step of the cascades of tumor invasion and metastasis. Therefore, it is necessary to further investigate the exosome-mediated intercellular communication network within the tumor microenvironment to elucidate the mechanisms of cancer metastasis. This review summarizes alterations in the tumor microenvironment at primary and metastatic sites during metastasis, encompassing processes such as epithelial-mesenchymal transition (EMT) induction, extracellular matrix (ECM) remodeling, immune suppression, and angiogenesis. In addition, we examine the role of exosomes in mediating tumor drug resistance and explore the clinical translational potential of exosomes in biomarker detection, drug delivery systems, and cancer vaccines.

## Introduction

Cancer metastasis—the process by which tumor cells spread from the primary site to distant organs, accounts for approximately 90% of cancer-related mortality^1^. Distant metastasis can be divided into two key stages: (1) Tumor cell invasion at the primary tumor site and migration to secondary sites;(2) the colonization and adaptation of these cells to the microenvironment of distant organs^2^. As the primary tumor proliferates, tumor cells acquire the ability to invade surrounding tissues, migrating from the stromal tissue of the originating organ and infiltrating the bloodstream. These cells become known as circulating tumor cells (CTCs) once they enter the circulatory system. Subsequently, CTCs strive to survive and infiltrate distant organs. Within the pre-metastatic niche of these organs, tumor cells gradually adapt and proliferate, forming metastatic lesions. This complex, multi-step process is known as the metastatic cascade^3^.

Research has demonstrated that complex paracrine and endocrine signaling between the tumor cells and their surrounding environment, which coordinates both the local and distant microenvironments, regulates the proliferation and metastasis of malignant tumors. The tumor microenvironment (TME) refers to the cellular environment in which the tumor resides, primarily composed of the extracellular matrix (ECM), various stromal cells (such as endothelial cells, immune cells, and fibroblasts), and numerous tumor-regulating factors^4^. The TME is intricate and continuously evolving, contributing to cancer cell heterogeneity, clonal evolution, and multi-drug resistance, ultimately driving tumor progression and metastasis^5^. Therefore, understanding the communication network between cancer cells, the original tumor site, and distant organs is critical for advancing cancer treatment.

Exosomes were first discovered in the 1980s as a form of extracellular vesicle released by most eukaryotic cells. They have a diameter of 30 to 150 nm and are made up of a membrane with a lipid bilayer that encloses complex molecular contents such as proteins, nucleic acids, and lipids^6^. Over the past decade, the role of exosomes in communication within the tumor microenvironment has attracted increasing attention. Exosomes mediate communication between tumor cells and stromal cells through three distinct pathways: (1) activation of target cell signals via receptor-ligand binding; (2) fusing with the membrane of the target cell to transport their contents; and (3) direct internalization by target cells^7^.

**Figure 1:**
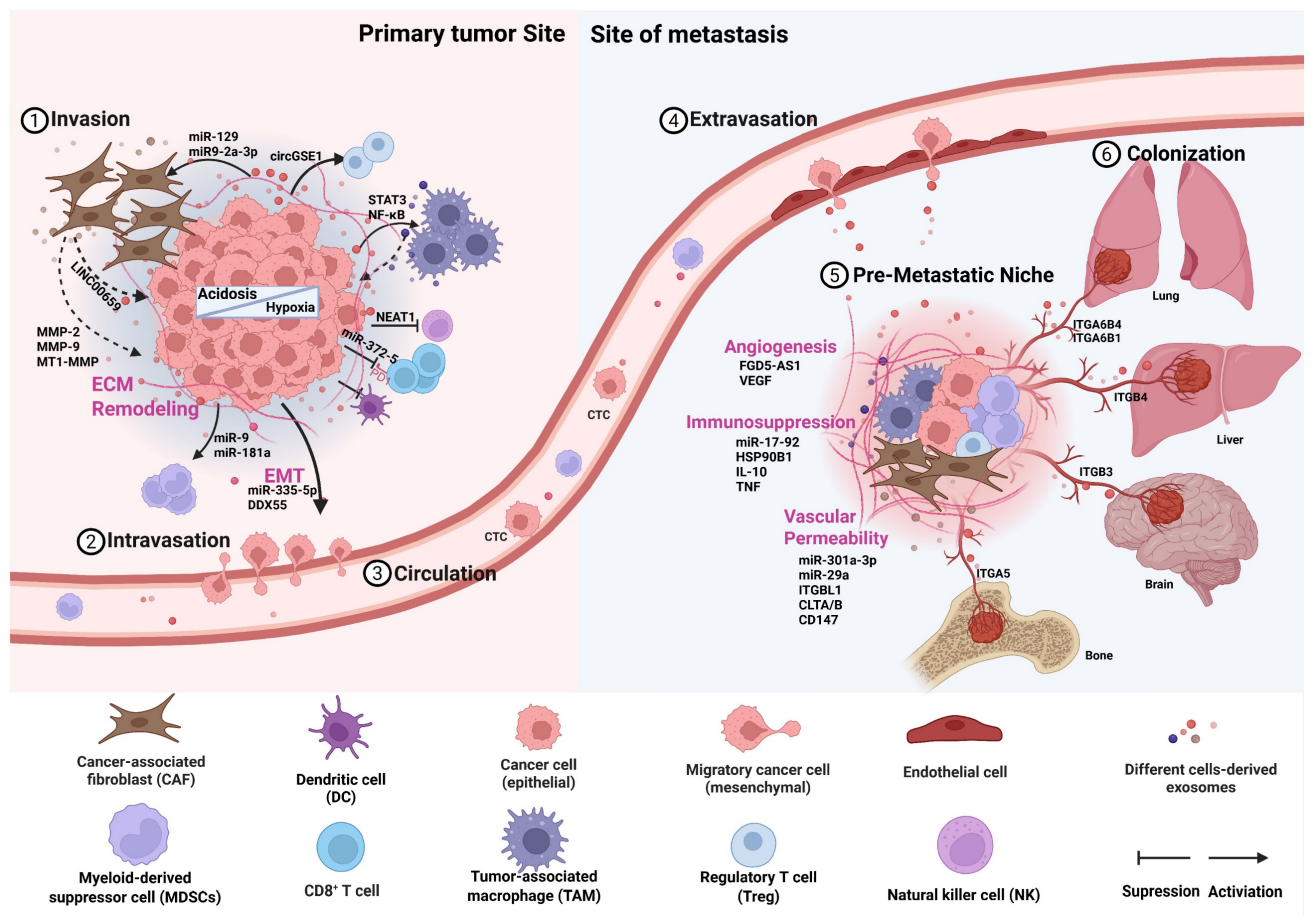
Exosome-mediated alterations in the tumor microenvironment at primary and metastatic sites. This schematic illustrates the cascade of events through which exosomes orchestrate microenvironmental changes supporting metastasis. The cascade is initiated within the primary tumor microenvironment (TME) by key stressors such as hypoxia and acidosis. Tumor cells and stromal cells release exosomes that promote local invasion and migration. These exosomes subsequently enter the circulation and travel to predetermined distant sites. At these sites, exosomes mediate the formation of a pre-metastatic niche by promoting angiogenesis, increasing vascular permeability, recruiting immunosuppressive cells, and remodeling the extracellular matrix (ECM). This prepared and hospitable niche enables the subsequent colonization and outgrowth of circulating tumor cells, ultimately leading to the formation of overt metastatic lesions. (Created with Biorender.com).

A rising amount of evidence reveals that exosomes play an important part in the multi-step process of tumor metastasis. This review explores how stressors, such as hypoxia and acidosis, regulate exosome synthesis and cargo sorting, along with particular alterations of the TME at both initial and distant sites during exosome-mediated tumor metastasis **(Figure 1)**. Furthermore, we examine the clinical significance of exosomes, including their roles in therapeutic resistance, biomarkers, drug delivery, and potential applications in tumor vaccine development.

## Regulation of exosome biogenesis and cargo by the TME

### Mechanisms of exosome biogenesis

Exosome biogenesis is closely linked to the endocytic system^8^. Early endosomes (EE) are generated by plasma membrane invagination and mature as late endosomes (LE), which further bud inward to produce intraluminal vesicles (ILVs). Endosomes harboring ILVs are known as multivesicular bodies (MVBs)^9^. Each step in this process involves the sorting of various proteins and bioactive small molecules. The secretion of ILVs relies on two distinct sorting mechanisms: the ESCRT (endosomal sorting complexes required for transport)-dependent pathway and the ESCRT-independent pathway^10^. ESCRT is a sophisticated protein machinery that serves a key function in exosome biogenesis via ILV formation. It is composed of four distinct protein complexes (ESCRT-0 to III) and associated proteins. Initially, the ubiquitin-binding subunit of ESCRT-0, Hrs, recognizes and sorts ubiquitinated proteins to endosomal membrane. ESCRT-0 then recruits ESCRT-I through connecting to tumor susceptibility gene 101. The ESCRT-I and -II complexes then facilitate the inward budding of the endosome around the cluster of ubiquitinated proteins. Finally, the ESCRT-III complex cleaves the buds to form ILVs, which are released from the MVB membrane^11^. Following fusion with the plasma membrane, the bulk of ILVs are discharged into the extracellular space as exosomes of diverse sizes and compositions.

### Regulation of exosome secretion by the tumor microenvironment

**Figure 2:**
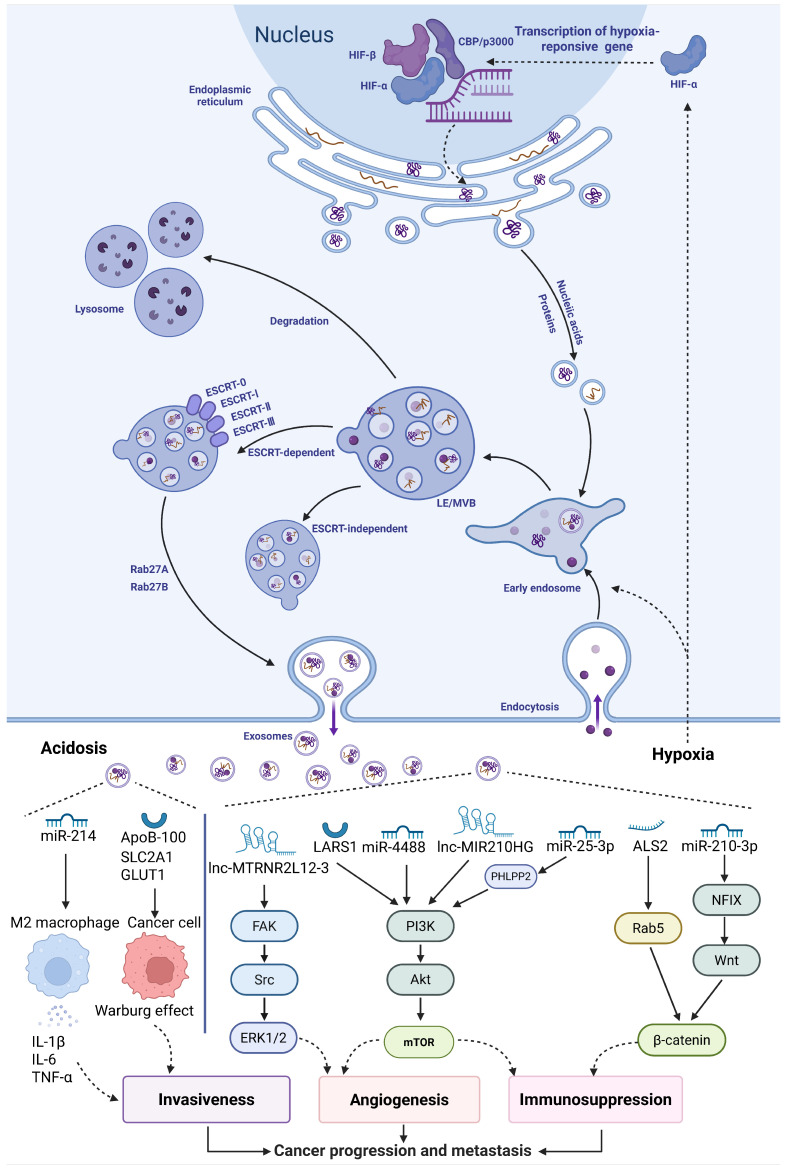
Mechanisms of exosome biogenesis and tumor microenvironment-driven cargo loading. The biogenesis of exosomes is intimately associated with the endosomal system, specifically through the inward invagination of the multivesicular body (MVB) membrane, which gives rise to intraluminal vesicles (ILVs). The fusion of ILV-containing MVBs with the plasma membrane, resulting in the extracellular release of exosomes. The key TME stressors of hypoxia and acidosis remodel the molecular cargo (e.g., proteins, miRNAs, mRNAs) of exosomes, thereby establishing a pro-tumorigenic communication network. (Created with Biorender.com).

Hypoxia, low pH, and nutrient deprivation are characteristics of the tumor microenvironment (TME), which creates an adaptive environment that encourages tumor growth. Tumor-derived exosomes, as distant messengers of these stress signals, show changes in secretion levels and functional properties, facilitating tumor progression, such as invasion, metastasis, and immune escape **(Figure 2)**. Notably, the concentration of exosomes in the blood of cancer patients is about twice that of healthy individuals (approximately 4×10^15 compared to about 2×10^15 particles), with this increase primarily driven by hypoxic and acidic conditions within the TME^12^.

Hypoxia is a major regulator of exosome secretion. Hypoxia is a major regulator of exosome secretion^13^. Early work by King et al. indicated that hypoxic breast cancer cells release more exosomes than cells under normoxic conditions, a process associated with HIF-1α activity^14^. Subsequent studies have demonstrated that hypoxia can regulate the expression and membrane localization of specific small GTPases (e.g., Rab27a, RAB22A) in a HIF-dependent manner, and these Rab proteins are key executors of exosome MVB transport, anchoring, and secretion^15^, ^16^. Acidosis is a metabolic consequence of hypoxia-driven glycolytic shift. According to the Warburg effect, hypoxia enhances glycolysis in tumor cells, and the accumulation of lactate and bicarbonate produced subsequently leads to the acidification of the tumor microenvironment^17^. Acidosis then serves as an evolutionary selection pressure, causing tumor cells to develop phenotypic plasticity that enables them to adapt to hypoxic and acidic environments. This adaptive change further exacerbates genomic instability in tumor cells and enhances their invasive capabilities^18^. Therefore, acidosis in the TME is closely associated with increased metastatic potential in various malignant tumors and poor prognosis in patients. In melanoma, this acidic environment has been shown to trigger a significant increase in exosome release. The potential mechanism may involve lowering membrane rigidity, which enhances membrane fusion and uptake efficiency, benefiting both exosome secretion and the re-endocytosis of exosomes by recipient tumor cells. Furthermore, Chenhao Jiang and colleagues discovered that tumor cells can take up extracellular lactate through monocarboxylate transporter 1 (MCT1), which facilitates the transport of multivesicular bodies (MVBs) to the plasma membrane, thereby enhancing exosome release. Mechanistically, lactate boosts the activity of the acetyltransferase p300, which catalyzes the acetylation modification of Rab7A.The acetylation of Rab7A inhibits its GTPase activity, thereby promoting the fusion of MVBs with the plasma membrane and exosome release^19^.

### Regulation of exosomal cargo by the tumor microenvironment

While increasing exosome secretion, the characteristic hypoxic and acidic conditions in the TME drive the increase in exosome heterogeneity and reprogram their cargo composition. For instance, under hypoxic stress, tumor cells selectively package and release exosomes containing the long non-coding RNA SNHG16. Once taken up by recipient cells, the SNHG16 delivered by exosomes acts as a competing endogenous RNA, adsorbing miR-132-3p and relieving its inhibition on the oncogene KIF5A. This signaling axis not only directly promotes cancer cell proliferation and stem cell properties but also induces tumor-associated macrophages to polarize towards the M2 phenotype, thereby jointly creating an immunosuppressive microenvironment^20^. In addition, the stress also regulates metabolic reprogramming through exosomes. Exosomes derived from hypoxic breast cancer cells display a distinct N-glycosylation profile, with significant increases in the expression of patterns such as H4N3F1S2, H3N3F1S0, and H7N4F3S2. These alterations help the cells adapt to hypoxic stress^21^. On the other hand, exosomes from hypoxic prostate cancer cells exhibit triglyceride accumulation, indicating that they may supply energy to recipient cells^22^.

Apart from hypoxia, acidosis also notably reprograms the cargo of exosomes. Andreucci et al. found that under *in vitro* simulated acidic conditions (pH 6.7±0.1), exosomes released by melanoma cells are rich in miR-214, which promotes microenvironmental inflammation and tumor cell migration by activating a pro-inflammatory macrophage phenotype and increasing vascular permeability^23^. Recent research also revealed that exosomes secreted by prostate cancer cells (PC-3AcT) pretreated in acidic medium undergo significant proteomic alterations, with 159 proteins showing differential expression. Among these, the expression level of apolipoprotein B-100 increased approximately 7.6-fold, and it can upregulate glucose transporter proteins SLC2A1/GLUT1 and other glycolysis-related genes, reprogramming normal PC-3 cells into the Warburg phenotype, thereby enhancing their survival and proliferation in the acidic microenvironment^24^. More information on the changes in exosome cargo and function induced by the hypoxic and acidic microenvironment can be found in **Table 1**. In conclusion, exosomes and the tumor microenvironment establish a dynamic bidirectional interaction network. On one hand, stress signals like hypoxia and acidosis not only quantitatively enhance exosome secretion levels but also qualitatively reprogram their cargo composition. On the other hand, these reprogrammed exosomes deliver specific signaling molecules (such as RNA, proteins, and metabolites), activate several pro-tumor pathways within recipient cells, and remodel the microenvironment into a state more favorable for tumor growth through immune suppression, angiogenesis, and other processes^20^, ^24^. However, the detailed regulatory mechanisms of selective cargo packaging in exosomes under hypoxia remain unclear. It is critical to study the specific features of exosomes generated from different hypoxic tumor tissues in order to provide novel therapeutic targets for dynamic monitoring of tumor progression.

## Exosomes in primary tumor progression

### Driving EMT and invasion

The importance of the epithelial-mesenchymal transition (EMT) in the development and metastasis of cancer has gained widespread recognition in recent years^25^. For metastasis to occur, before tumor cells may penetrate the basement barrier and invade the blood stream, they must develop robust migratory and invasion abilities, facilitating their spread to distant locations. This critical step is largely driven by EMT. Mechanistically, EMT causes cancer cells to produce less E-cadherin and more vimentin, leading in cell-cell junction disintegration, loss of apical-basal polarity, and improved migratory and invasion capabilities^26^. This provides the foundation for metastasis and resistance to chemotherapy and radiotherapy. Exosomes facilitate the initiation of EMT and endow tumor cells with invasive properties by modulating various oncogenic signaling pathways.

According to a number of studies, tumor-derived exosomes are essential for controlling the EMT process^27^. For example, exosomes derived from CRC can deliver miR-335-5p, directly targeting and inhibiting the expression of RAS p21 protein activator 1 (RASA1). The p120RasGTPase-activating protein encoded by RASA1 is a key negative regulator of the RAS/MAPK pathway, and its downregulation leads to sustained activation of this pathway, driving EMT and ultimately promoting tumor cell invasion and metastasis^28^. Studies have shown that upregulation of DEAD-box helicase 55 (DDX55) is associated with prognosis in hepatocellular carcinoma (HCC) patients. Exosomes derived from HCC activate the PI3K/Akt/GSK-3β/β-catenin pathway in HCC, driving malignant progression of the disease. Mechanistically, DDX55 interacts with BRD4, together occupying the promoter region of the PIK3CA gene, thereby activating the PI3K/Akt signaling pathway. The activated PI3K/Akt signaling pathway inactivates GSK-3β via phosphorylation, thereby inhibiting GSK-3β-mediated phosphorylation and degradation of β-catenin. This series of events leads to increased stability of β-catenin in both the cytoplasm and nucleus, subsequently inducing transcriptional reprogramming of its downstream target genes, ultimately promoting cell cycle progression and EMT^29^. Exosomes derived from non-tumor cells also trigger the initiation of EMT. For instance, cancer-associated fibroblasts (CAFs) deliver the lncRNA LINC00355 to colorectal cancer cells through exosomes. LINC00355 serves as a molecular sponge for miR-34b-5p, alleviating its inhibition of the CRKL gene and leading to upregulation of CRKL protein expression. Importantly, the CRKL gene, a critical signaling molecule, regulates the Ras/MAPK signaling pathway through its SH2 and SH3 domains, thereby collaboratively enhancing cancer cell proliferation and EMT progression^30^. Tumor-associated macrophages with a pro-invasive phenotype promote tumor progression by secreting exosomes. In both *in vivo* and *in vitro* experiments, exosomes carrying high levels of miR-95 act as tumor initiators by activating the target gene JunB in prostate cancer cells, which promotes cancer proliferation, invasion, and EMT^31^.

However, the role of exosomes in tumor progression is not unidirectional. Their functional output shows a complex duality: exosomes can drive invasion, yet under specific conditions, they may exert a tumor-suppressive effect. This stark contrast in function is primarily determined by the following factors acting together:(1) Differences in pathological or physiological inducing factors in the body. (2) The different cell origins determine the functional differences^32^, ^33^. For instance, the gastric-specific protein GKN1, which is produced and secreted by gastric mucosal epithelial cells, can act as an exosome cargo to exert tumor-suppressive effects. Mechanistically, the BRICHOS domain of GKN1 binds strongly to HRas, preventing GTP-bound HRas from interacting with its downstream effector proteins, b-Raf and c-Raf. This action inhibits the activation of the Ras/Raf/MEK/ERK kinase signaling pathway, which significantly suppresses the expression of several EMT-related proteins^34^. Furthermore, exosomes originating from mesenchymal stem cells deliver miR-200a, suppressing ZEB1 and increasing E-cadherin expression, thus blocking the EMT process^35^. Exosomes derived from bone marrow mesenchymal stem cells can exert tumor-suppressive effects by delivering let-7i. The exosomal let-7i inhibits the transcription factor KDM3A, downregulating the expression of its target gene DCLK1, thereby relieving DCLK1's suppression of FXYD3, ultimately effectively curbing the proliferation, migration, and invasion of lung cancer cells^36^. In summary, the function of exosomes depends on the specific molecular cargo they carry, which is dynamically regulated by the microenvironment and the cell of origin. Understanding how these cargoes determine the functional differences of exosomes is crucial for elucidating their contradictory roles in cancer, and it also provides precise targets for exploiting or modulating exosomal function.

### Inducing ECM degradation

The extracellular matrix (ECM) is a multi-molecular network composed of a variety of proteins, including collagen, fibrin, proteoglycans, and elastin, that is essential in tumor occurrence, development, and metastasis. Tumor cells at the primary site must extensively remodel the ECM to establish a structural and bioactive environment conducive to invasion and dissemination^37^.

Exosomes facilitate ECM protein degradation by activating or delivering matrix metalloproteinases (MMPs) and encouraging the development of invasive pseudopodia^38^. MMPs are a class of soluble or membrane-bound proteases essential for ECM remodeling, expressed by cancer cells and non-tumor cells (primarily CAFs and endothelial cells). Exosomes produced after co-culturing fibroblasts with thyroid tumor cells activate MMP-2 to promote ECM component degradation^39^. Similarly, TNBC-derived exosomes activate MMP-2 and MMP-9, promoting the formation of invasive pseudopodia to facilitate ECM degradation^40^. Exosomes also transfer MMP-2 to convey the invasive pseudopodia activity in tumor cells^41^. Therefore, a synergistic relationship exists between exosomes and invasive pseudopodia. A recent study has demonstrated for the first time that tethered exosomes play a functional role in tumor migration. Exosomes containing MT1-MMP are anchored to the surface of migrating cells by fibronectin, which enhances local ECM degradation and promotes invasion and migration^42^.

Cancer-associated fibroblasts (CAFs) are key components of the tumor stroma, remodeling the ECM by depositing ECM proteins, secreting growth factors, and contracting the ECM. Multiple studies have shown that s cancer-derived exosomes can trigger the differentiation of normal fibroblasts (NFs) and MSCs into CAFs, thereby enhancing invasion and migration^43^. For example, cisplatin-treated latent lung adenocarcinoma (LUAD) cells that release exosomes that carry ITGB6 to fibroblasts. This causes CAF differentiation by activating the TGF-β and KLF10 pathways^44^. Exosomal miR-12961 and miR-92a-3p promote the conversion of NFs to CAFs by targeting the MT1G/AKT and KLF4/CH25H axes^45^, ^46^. Furthermore, Hepatic stellate cells (HSCs) are converted into CAFs by HCC-derived exosome miRNA-21, which inhibits PTEN and activates the downstream PDK1/AKT pathway^47^.

It is noteworthy that exosomes secreted by CAFs can also act on tumor cells, promoting tumor progression^48^. CAF-derived exosomes containing LINC00659 interact with miR342-3p to enhance ANXA2 expression, promoting CRC proliferation, invasion and migration^49^. CAF-derived exosomes containing miR-181b-3pand miR-345-5p promote tumor progression^50^, ^51^. The transformation and activation of CAFs are associated with tumor progression and poor prognosis of various cancers, and exosomes can serve as a potential target to block the interaction between CAFs and the TME, thereby inhibiting CAF-mediated stromal remodeling. These studies suggest that exosomes regulate the extracellular matrix and stromal cells (especially fibroblasts), creating conditions conducive to further tumor cell metastasis.

### Mediating immunosuppression

Tumor cells modify immune cell (including lymphocytes, macrophages, myeloid-derived suppressor cells, and granulocytes) in order to avoid immune system identification and elimination, thereby promoting malignant tumor proliferation. As mediators of tumor-stroma crosstalk, exosome-mediated immune suppression operates through a multi-layered regulatory network.

Macrophages are typically categorized into pro-inflammatory M1 and immune-suppressive M2 types. Clinical and experimental data indicate that M1-polarized macrophages exhibit anti-tumor activity, while M2-polarized macrophages promote tumor growth. Tumor-associated macrophages (TAMs) typically exhibit an M2-like phenotype and are crucial for tumor development and growth^52^. Pancreatic neuroendocrine tumor cells produce exosomes rich in CEACAM5 in a hypoxic microenvironment, inducing M2 polarization of TAMs by activating the MAPK signaling pathway^53^. Exosomes from glioma and pancreatic cancer cells containing circ-001422 and lncRNA FGD5-AS1 have been shown to mediate STAT3 acetylation by interacting with p300, thereby activating the STAT3/NF-κB pathway to induce M2 macrophage polarization^54^, ^55^. Furthermore, miR-200b is significantly overexpressed in ovarian cancer-derived exosomes. This microRNA downregulates KLF6 expression, thereby inducing M2 polarization, which in turn facilitates the proliferation and invasion of ovarian cancer cells^56^.

Exosomes from various sources can regulate the expression of PD-L1, thereby directly or indirectly suppressing T cell function. PD-L1 is present on the surface of various tumor cells and immune cells in the TME, and its binding to PD-1 on T cells inhibits T cell activation, making it a crucial target for immune checkpoint inhibitors (ICIs). CAF-derived exosomes containing circHIF1A and zinc finger protein ZNF250 stabilize PD-L1 expression in HCC cells, thereby inducing immune escape^57^, ^58^. CRC-derived exosomes activate the PTEN/AKT/NF-κB signaling to enhance TAM-mediated suppression of CD8^+^ T cell. More specifically, knockdown of exosomal miR-372-5p decreases PTEN expression in macrophages, leading to an increase in pAKT/AKT and p-P65/P65 levels, downregulating PD-L1 expression, and suppressing cancer cell proliferation and progression^59^. Furthermore, nasopharyngeal carcinoma cells directly release exosomes carrying PD-L1 protein, which connect to the PD-1 on CD8+ T cells, directly inhibiting their cytotoxic function^60^.

Exosomes promote immune evasion by regulating the number and function of natural killer (NK) cells and regulatory T cells (Tregs). For example, exosomes derived from multiple myeloma cells carry lncRNA NEAT1, which downregulates the pre-leukemia transcription factor PBX1 and inhibits NK cell activity, thereby promoting immune evasion in multiple myeloma^61^. Conversely, exosomes from HCC containing circGSE1 activate and induce Tregs, suppressing effector T cell function and creating a tumor microenvironment conducive to immune escape. Mechanistically, TGFBR1 is the receptor for TGFβ1, and Smad3 is an important substrate for TGF-β, contributing to the expansion of Tregs. circGSE1 functions as a sponge for miR-324-5p, activating TGFBR1 and Smad3^62^.

Exosomes facilitate a tumor-friendly microenvironment by inducing the activation and expansion of myeloid-derived suppressor cells (MDSCs), thereby promoting tumor metastasis. MDSCs consist of immature myeloid cells (IMCs) and exhibit potent immunosuppressive activity. Exosomal miR-9 and miR-181a stimulate the growth of early MDSCs via stimulating the JAK/STAT signaling pathway^63^**.** Glioma-derived exosomal lncRNA Agap2-As1 increases MDSC secretion of TGF-β1 by targeting miR-486-3p, thereby participating in immune cell signaling^64^. Overall, these findings systematically reveal the multi-pathway synergistic roles of exosomes in tumor immune regulation. Refining the exosome-mediated immunosuppressive network will aid in the development of immune therapeutic targets and enhance understanding of mechanisms of treatment resistance.

## Exosome-mediated pre-metastatic niche formation

### Deciphering the code of organotropic metastasis

**Figure 3:**
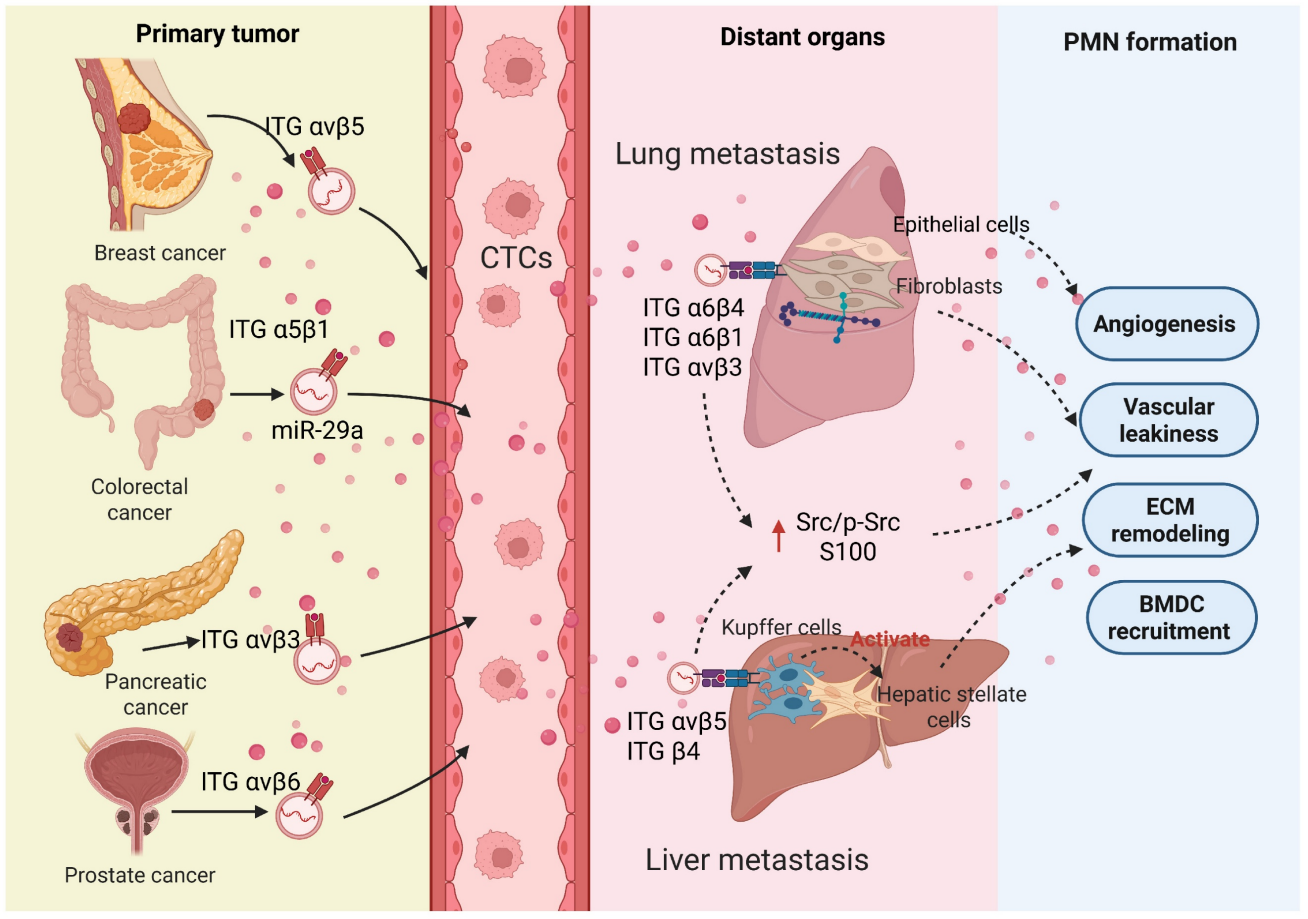
Mechanism of different exosomal integrins-mediated organ-specific metastasis. Primary tumor cells (e.g., from breast, colorectal, pancreatic, and prostate cancers) release exosomes that travel to distinct distant organs (such as liver and lung) via integrin-specific trafficking and target resident cells (e.g., fibroblasts and Kupffer cells). Subsequently, they promote the establishment of the pre-metastatic niche by enhancing vascular permeability, stimulating angiogenesis, remodeling the extracellular matrix (ECM), and recruiting bone marrow-derived cells (BMDCs). (Created with Biorender.com).

The "seed and soil" hypothesis proposes that there is a selective interaction between tumor cells (the seed) and distant organs (the soil), explaining why tumor cells preferentially metastasize to certain organs (e.g., lungs, liver, brain, and bones)—a phenomenon known as organ-specific metastasis^2^, ^65^. Growing research indicates that, apart from the intrinsic genetic determinants of tumor cells, organ-specific metastasis is closely linked to integrins present on the surface of exosomes **(Figure 3)**.

Integrins (ITG)are a class of ubiquitously present transmembrane adhesion receptors, serving as key bridges connecting the extracellular matrix to the intracellular cytoskeleton. They precisely regulate cell behavior through bidirectional signaling: the extracellular domain recognizes and binds extracellular matrix proteins, while the intracellular domain interacts with various cytoskeletal proteins and signaling molecules^66^. A study by Ayako Hoshino and colleagues suggests that the uptake of tumor exosomes by resident cells in different organs depends on the specific integrin expression patterns on their surface. For example, exosomal integrins α6β1 and α6β4 promote lung metastasis of breast cancer by activating Src phosphorylation and inducing pro-inflammatory S100 gene expression. Exosomes containing αvβ5, on the other hand, are specifically absorbed by hepatic Kupffer cells, promoting liver metastasis^67^. Similarly, studies have shown that high expression of exosomal ITGA6 and ITGB4 in colorectal cancer is linked to lung organotropism and significantly enhances the proliferation and tubulogenesis of HUVECs^68^. Interestingly, highly invasive cancer cells tend to secrete higher levels of integrins, and these exosomal integrins remodel the invasive phenotype of target cells, creating favorable conditions for metastasis. In epithelial ovarian cancer (EOC), high expression levels of exosome-derived ITGA3 are closely associated with liver metastasis^69^. ITGA3 upregulates the expression of S100A7 in recipient cells, thereby activating the p-ERK/ERK signaling pathway, while knocking down S100A7 reverses this activation effect. The activation of this signaling axis significantly boosts cancer cell migration, invasion, and their colonization in the liver^70^. A study found that ITGA5 and ITGB1 are highly expressed in the serum and ascites of EOC patients and co-localize with asparagine endopeptidase (AEP). When HPMCs take up exosomes carrying the ITGA5/ITGB1/AEP complex, the FAK/Akt/Erk pathway is activated, thereby laying the foundation for peritoneal metastasis^71^. Different types of integrins carried by exosomes that induce organ-specific metastasis and promote PMN formation are depicted in **Table 2.** These findings collectively highlight the central role of exosomal integrins in mediating organ-specific metastasis. The mechanism may involve two aspects: one is directly enhancing the invasive ability of tumor cells at the primary site, and the second is actively remodeling the microenvironment at the site by selective uptake of exosomes by resident cells of specific distant organs, thereby guiding and promoting metastatic colonization. Future research should focus on elucidating the integrin profiles of metastatic lesions, aiming to provide new targets and decision-making frameworks for precision treatment.

### Modulating angiogenesis and vascular permeability

To guarantee the nutrient supply for rapid tumor growth in distant organs, PMN triggers angiogenesis and increases vascular permeability. Normally, endothelial cells are connected by adhesion molecules and tight junctions to limit the passage of proteins and cells, thereby maintaining the vascular barrier. Soluble factors and exosomes secreted by tumor cells impair endothelial cell junctions, thereby increasing vascular permeability and allowing the entry of immune cells, stromal cells, and tumor cells into the PMN^72^.

The levels of non-coding RNAs (ncRNAs) in tumor-derived exosomes are crucial for vascular leakage. KLF, a zinc finger transcription factor, regulates endothelial adhesion. Exosomes transport miR-3157-3p and miR-29a to endothelial cells, inhibiting KLF2 and KLF4, which leads to the upregulation of angiogenesis factors (VEGF, MMP2/MMP9) and downregulation of tight junction proteins, thus enhancing vascular permeability in metastatic NSCLC and CRC^73^, ^74^. Studies have found that the expression of lncRNA FGD5-AS1 in serum exosomes from metastatic thyroid cancer (TC) patients is higher than in adjacent tissues. FGD5-AS1 promotes vascular leakage by regulating the miR-6838-5p/VAV2 pathway^75^.

Exosomes promote angiogenesis and remodel the microvascular niche. The heparin-binding VEGF on the exosomal surface has signaling capacity, promoting endothelial cell migration and vascular formation by binding to and activating the tyrosine kinase receptor VEGFR2, without being neutralized by bevacizumab^76^. Interestingly, research found that treatment with exosomes enriched in GTF2H2 derived from HCC decreased VEGFR and MMP2/9 expression in human umbilical vein endothelial cells (HUVECs), inhibiting endothelial viability and tumor migration^77^. Likewise, silencing aminopeptidase N from RCC-derived exosomes reduces endothelial gap formation and angiogenesis in the bone marrow, inhibiting CTC migration to the bone marrow microenvironment^78^. These findings suggest that the mechanisms by which exosomes mediate angiogenesis could serve as potential molecular targets for tumor inhibition. Chronic inflammation is a key driver of tumor progression and metastasis. Exosomes facilitate the release of pro-inflammatory factors by resident cells in distant organs, enhancing vascular permeability. CRC releases exosomes enriched with ITGBL1 into circulation, activating resident fibroblasts in lung and liver metastatic tumors, promoting the secretion of pro-inflammatory cytokines such as IL-6 and IL-8, and inducing the formation of an inflammatory microenvironment^79^.

### ECM remodeling in distant organs

During PMN formation, tumors deposit new extracellular matrix components through soluble factors, exosomes, and direct cell-to-cell interactions, which alter the matrix composition^80^. Multiple ECM molecules, such as fibronectin, osteopontin, and multifunctional proteoglycans, specifically deposit in the PMN, providing substrates for the anchoring of CTCs. Meanwhile, the ECM in the PMN is rich in MMPs and LOX family proteins, which, by catalyzing collagen crosslinking, not only enhance matrix stiffness but also promote the homing of bone marrow-derived cells and CTCs to this site^81^, ^82^.

In the PMN of the liver, fibronectin deposition mediated by activated stromal fibroblasts can be observed^81^. As the primary stromal cells in the liver, activated hepatic stellate cells (HSCs) can transform into myofibroblasts and play a key role in cancer progression by secreting cytokines and ECM components. Exosomes participate in the activation of HSCs through various mechanisms. For example, exosomes derived from pancreatic ductal adenocarcinoma (PDAC) deliver the CD44v6/C1QBP complex to the HSC membrane, triggering the phosphorylation of IGF-1signaling molecules and subsequently activating HSCs^83^. Similarly, exosomal miR-188-3p derived from colorectal cancer (CRC) cells can activate HSCs by targeting PHLPP2^84^. Exosomes derived from PDAC can be taken up by Kupffer cells, stimulating the secretion of TGF-β, which in turn activates HSCs, induces significant deposition of fibronectin, and promotes the recruitment of macrophages and neutrophils to the PMN^85^. Additionally, during the early metastatic stage of salivary duct carcinoma, the lungs are the most common site of metastasis. During this process, exosomes from CAFs are taken up by lung fibroblasts via integrin α2β1, thereby activating the TGF-β signaling pathway within the cells and inducing the overexpression of POSTN^86^.

Cytokines resident in or newly secreted by the ECM are key drivers in recruiting monocytes and macrophages to the microenvironment. Primary tumors release exosomes rich in integrin β-1-like protein into the circulatory system. These exosomes can directly bind toTNFAIP3, activating the NF-κB signaling pathway, which transforms lung fibroblasts and HSCs into an activated phenotype. Activated cells then secrete pro-inflammatory cytokines such as IL-6 and IL-8, inducing the formation of pro-metastatic ECM^79^. Monocyte chemoattractant protein-1 (MCP-1) and stromal cell-derived factor-1 (SDF-1) play particularly crucial roles in mediating the specific recruitment of monocytes and macrophage polarization. For example, exosomes carrying CXCR4 can bind to SDF-1α expressed on the surface of lymphatic endothelial cells (LECs), thereby enhancing the secretion of MMP-9, MMP-2, and vascular VEGF-C by LECs. These factors collectively promote LEC proliferation and lymph angiogenesis, thereby creating favorable conditions for the colonization of liver cancer cells in lymph nodes^87^. Similarly, exosomal miR-122-5p derived from breast cancer cells can directly target mitogen-activated protein kinase phosphatase-2 (MKP-2) in lung fibroblasts, upregulating the secretion of MCP-1 and SDF-1, thereby driving the lung metastasis of breast cancer^88^. A detailed analysis of the interactions between exosomes and the molecular components of the ECM is crucial for elucidating the mechanisms of ECM remodeling mediated by exosomes and for developing targeted intervention strategies based on these insights.

### Recruitment and education of bone marrow-derived cells in immunosuppression

Once exosomes from circulation arrive at distant organs, they recruit and educate bone marrow-derived cells (BMDCs), converting them into immune-suppressive cells that aid tumor progression, thus creating a safe microenvironment for tumor cells. These cells contribute to tumor cell evasion of immune surveillance, thereby establishing an immune-suppressive microenvironment, a key feature in the formation of the PMN^89^.

Exosomes trigger the secretion of immune-suppressive cytokines, modulating the local microenvironment at potential metastatic sites. In the gastric cancer peritoneal metastasis model, exosome-induced immune-suppressive PMN is predominantly driven by macrophages. Mechanistically, gastric cancer cells enhance exosome secretion via the METTL3-m⁶A axis. Exosomes transfer miR-17-92 to peritoneal macrophages, inhibiting SRC kinase signaling inhibitor 1, activating SRC proto-oncogene signaling, elevating IL-10 and TNF levels, and reducing IL-1 and IL-6 levels, thereby creating a pro-metastatic immune microenvironment in the peritoneum^90^. Additionally, colorectal cancer-derived exosome HSP90B1 can polarize M1 macrophages to the M2 phenotype and reduce CD8+ T cell activity, promoting liver metastasis^91^.

Tumor-derived exosomes are essential for attracting MDSCs. Exosomes from PDAC travel through the bloodstream to the pre-metastatic liver microenvironment, delivering tRF-GluCTC-0005 to hepatic stellate cells, activating the WDR1/YAP signaling axis, recruiting MDSCs, and establishing immune suppression, which then promotes liver metastasis^92^. In addition, during the early stages of tumor development, the migration of bone marrow-derived neutrophils contributes to the immune suppression within the host organ. Primary tumors secrete exosomes that express Lin28B, which promote neutrophil recruitment and polarization to the N2 phenotype, thus creating an immune-suppressive microenvironment characterized by upregulated PD-L2 expression and cytokine dysregulation^93^.

## Clinical implications and translational applications

### Exosome-mediated drug resistance

Drug resistance poses a critical challenge to cancer treatment. Over time, tumor cells develop resistance through genetic and phenotypic alterations within the cells, or as a result of interactions with surrounding cells^94^. Recent studies have found that exosomes mediate the interaction between cancer cells and stromal cells in the tumor microenvironment, thus increasing the complexity of resistance mechanisms and promoting tumor progression^95^.

Exosomes deliver bioactive molecules that transfer drug resistance to sensitive cells, facilitating the spread of the resistant phenotype in tumor populations^96^. Paclitaxel, a commonly used first-line chemotherapy agent for breast cancer, is one such example. Paclitaxel-resistant breast cancer cells and their exosomes express higher levels of miR-99b-3p compared to sensitive cells and their exosomes. Further studies confirmed that miR-99b-3p promotes AKT/mTOR phosphorylation by targeting the protein phosphatase 2 catalytic subunit α, thereby conferring paclitaxel resistance to sensitive cells^97^. Similarly, exosomes from breast cancer cells resistant to the mTOR inhibitor rapamycin were shown to significantly activate MAP kinase and AP-1 signaling in sensitive cells, contributing to drug resistance. Notably, DNA methyltransferase 3αmay play a role in suppressing rapamycin resistance^98^. Additionally, Qin et al. demonstrated how pancreatic cancer cells use exosome miR-31-5p to regulate the Hippo/SPARC pathway in pancreatic stellate cells, promoting chemotherapy resistance in pancreatic cancer cells^99^.

Exosome-mediated chelation and enhanced drug efflux in tumor cells can significantly reduce or even eliminate the efficacy of chemotherapy drugs by directly encapsulating and expelling them^95^. For example, in ovarian cancer, the cisplatin content in exosomes released by resistant tumor cells is notably higher than in sensitive cells^100^. Additionally, Liu et al.'s review provides a comprehensive summary, highlighting that many chemotherapy drugs (e.g., enzalutamide, cisplatin, 5-Fu, paclitaxel) can be actively transported out of cells through this mechanism, thereby lowering intracellular drug concentrations in tumor cells^96^. In conclusion, these research findings show exosomes' potential as a target for overcoming medication resistance. Understanding exosome-mediated resistance mechanisms will aid in the development of more effective cancer treatments.

### Exosomes as potential biomarkers

Current cancer detection methods, such as imaging, pathological diagnosis, and tumor markers, often lack sufficient sensitivity for early-stage lesion detection. Exosomes, with their stable lipid bilayer and low immunogenicity, play distinct roles throughout tumor metastasis. As a result, tumor-specific proteins and RNA from exosomes have the potential to serve as biomarkers for clinical diagnosis, prognosis, and metastasis risk assessment^101^. For instance, Nakamura et al. conducted a whole-genome analysis of blood specimens and demonstrated that specific exosomal miRNAs could be used as biomarkers, improving the diagnostic accuracy of early-stage pancreatic ductal adenocarcinoma^102^. More information on exosomes as biomarkers for diagnosis and prognosis are depicted in **Table 3**.

It is noteworthy that during the cascade of tumor metastasis, the dynamic evolution of exosomal cargo profiles can be used to infer the real-time progression stage of the tumor. For example, in the early stages of tumorigenesis and local progression, exosome-mediated miR-720 targets and inhibits the expression of StarD13, promoting the proliferation and inhibiting apoptosis of hepatocellular carcinoma cells. Detection of exosomal miR-720 in serum can enable early diagnosis of small hepatocellular carcinoma (< 2cm), showing superior performance compared to AFP or DCP^103^. As the tumor progresses to the invasive and metastatic stages, there is a significant difference in the exosomal cargo profiles between metastatic and non-metastatic patients. has-let-7f-5p is expressed significantly higher in the serum exosomes of pancreatic cancer patients with distant metastasis compared to non-metastatic patients, and can be used to enhance the early detection and risk stratification of pancreatic cancer metastasis^104^. In contrast to patients without metastatic disease, plasma-derived EV miR-6084 levels are significantly reduced in colorectal cancer patients with liver metastasis^105^. Exosome-mediated drug resistance can serve as a dynamic indicator for monitoring treatment response and adjusting strategies in a timely manner. For example, exosomal miR-9-5p derived from CAFs targets CREBRF to activate the MAPK pathway, mediating cisplatin resistance in osteosarcoma and being associated with poor prognosis^106^. By establishing a model linking the multi-omics exosomal profile (including integrin membrane proteome, RNA profile, and proteome) with clinical staging, it is expected that future cancer staging will surpass traditional imaging and pathology-based methods, enabling dynamic, real-time, and non-invasive tumor staging.

### Engineered exosomes as drug delivery systems

Unlike synthetic nanoparticles, exosomes are natural nanocarriers with inherent targeting capabilities, equipped with various surface proteins that facilitate participation in cell communication. Additionally, exosomes possess biocompatibility, biodegradability, and the ability to cross multiple biological barriers, providing them with a distinct advantage in drug delivery^107^.

Exosome-based therapies, particularly those utilizing tumor suppressor miRNAs, hold significant potential for cancer treatment. For instance, exosomes loaded with miR-29a-3p effectively reduce collagen I levels to reshape the ECM of the PMN and inhibit lung cancer metastasis^108^. Similarly, animal studies have demonstrated that exosome-miR-338-3p inhibits the proliferation of gastric cancer cell lines (MKN45 and HGC27) and their adhesion to mesothelial cells, indicating its therapeutic potential in preventing the peritoneal spread of advanced gastric cancer^109^. However, it is important to note that due to the complexity of the *in vivo* tumor microenvironment, translating *in vitro* efficacy to significant *in vivo* outcomes remains a major challenge, potentially impacting the therapeutic effectiveness of miRNA-loaded exosomes.

Exosomes are capable of encapsulating a variety of drugs, enhancing their therapeutic efficacy and improving cancer treatment outcomes. Zhou et al. developed a liposome/macrophage-derived exosome mixed delivery system that simultaneously loads the phototherapeutic agent IR780 and heme, significantly enhancing the phototherapy effect in skin melanoma^110^. Similarly, Zhang's team created a bone marrow mesenchymal stem cell-derived exosome nanomedicine loaded with galectin-9 siRNA, DOGEM, and indocyanine green, which enhances the tumor-killing ability of CD8⁺ T cells, reduces the proportion of regulatory T cells (Tregs), and improves the immune-suppressive microenvironment in pancreatic cancer^111^. In the case of refractory KRAS-mutant colon cancer, co-delivery of siRNA, 3-bromopyruvate (3BP), and cetuximab (CTX) successfully suppressed KRAS oncogene expression and induced cancer cell apoptosis^112^. **Table 4** depicts a further array of cancer treatment strategies founded on exosome delivery platforms. These studies indicate that therapeutic strategies should be aligned with the cellular origin of exosomes^113^. For instance, tumor cell-derived exosomes, leveraging their innate tropism for homologous cancer cells, are often employed to deliver nucleic acid therapeutics (e.g., siRNA, miRNA) back to tumor sites. In contrast, MSC-derived exosomes, capitalizing on their superior biocompatibility and low immunogenicity, are frequently utilized as carriers for chemotherapeutic drugs or targeted siRNAs. Consequently, when engineering novel exosome-based drug delivery systems, close attention must be paid to their source-specific properties in order to develop potent and personalized cancer treatment strategies.

### Harnessing exosomes for cancer vaccines

Cancer vaccines represent an emerging form of immunotherapy that activate the immune system by delivering large quantities of tumor antigens alongside adjuvants, thereby enhancing immune responses and improving the effectiveness of immunotherapy^114^. Due to their superior carrier properties and specific immune activation capabilities, exosomes can function as both adjuvants and antigens to induce potent antitumor immune responses. Currently, exosome-based cancer vaccines can be classified into three types: (1) Whole exosomes derived from tumor cells and dendritic cells (DCs), which directly alleviate immune suppression and enhance immunostimulatory characteristics; (2) Exosome-activated dendritic cell vaccines; and (3) Vaccines composed of exosome membrane-loaded nanomaterial cores^115^.

In mouse models and human breast cancer organoids, breast cancer-derived exosomes loaded with human neutrophil elastase and histone (a TLR3 agonist) exhibit antitumor and immune-activating activity. This vaccine significantly enhances antigen cross-presentation by cDC1 and stimulates the generation of tumor-reactive CD8⁺ T cells^116^. Furthermore, induced pluripotent stem cells (iPSCs) are capable of producing a variety of tumor antigens, thereby triggering antitumor responses and inhibiting tumor growth. When iPSC-derived exosomes are incubated with dendritic cells (DCs) and pulsed to form a vaccine, they induce tumor cell killing in various cancers, including melanoma, lung cancer, breast cancer, and colorectal cancer. Notably, in mouse models, this vaccine significantly suppressed melanoma growth and generated long-lasting immune memory against tumor cells, preventing relapse^117^.

Nevertheless, tumor-derived exosome vaccines currently do not provide adequate antitumor immune responses^115^. The effectiveness of exosome vaccines relies on the immunosuppressive status of cancer patients, and exosomes themselves have weak immunogenicity and limited immune-suppressive functions^118^. Moreover, clinical trials of exosome-based anticancer vaccines are still in the early stages and require further detailed and comprehensive studies to establish their efficacy.

### Clinical translation: perspectives and challenges

**Figure 4:**
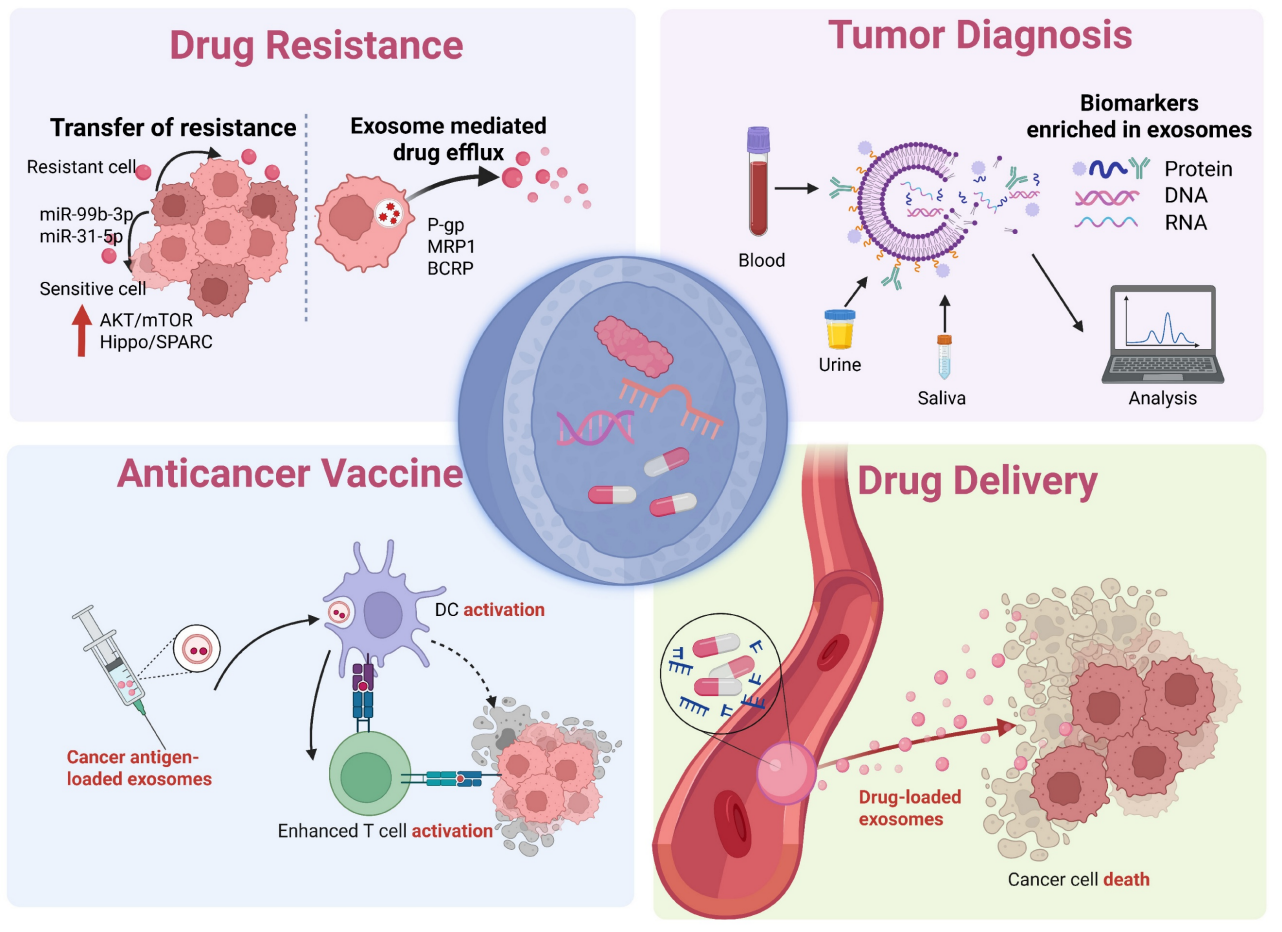
Clinical applications of exosomes. The multifaceted role of exosomes in therapeutic innovation, including mediating tumor drug resistance and their clinical translational potential in biomarker detection, drug delivery, and cancer vaccines. (Created with Biorender.com).

We have discussed the mechanisms by which exosomes mediate tumor drug resistance and their applications in liquid biopsy for early diagnosis, treatment efficacy monitoring, and metastasis prediction. Furthermore, we have highlighted their potential as drug delivery vehicles and cancer vaccines in the field of immunotherapy **(Figure 4)**. Despite the significant potential of exosome therapies in cancer treatment, several challenges remain in their practical application, necessitating further research and solutions.

The inherent heterogeneity of exosomes is a core bottleneck limiting their translation into clinical applications. This heterogeneity is reflected in multiple dimensions, including their physical size, cargo composition, membrane surface molecular markers, and the source of the producing cells. The heterogeneity of exosomes primarily arises from: (1) biological source heterogeneity, (2) heterogeneity due to physiological and pathological conditions, and (3) subpopulation heterogeneity^119^. This complexity has led to a lack of a widely accepted standardized classification framework in the academic community, which has posed multiple challenges in both research and translation.

Exosomes and their contents, as biomarkers, show significant potential in the early diagnosis, precise staging, and prognostic evaluation of various cancers. However, there is still a lack of an integrated technological platform capable of simultaneously achieving efficient exosome isolation, high-purity enrichment, and detailed molecular characterization^120^. Current mainstream methods (such as differential centrifugation and size exclusion chromatography) often struggle to balance high recovery rates, high purity, and resolution of functional subpopulations, with poor batch-to-batch reproducibility. Although emerging microfluidic technologies facilitate high-throughput analysis, quality control and standardized procedures for their large-scale application have yet to be established^121^. In the realm of engineered drug delivery, the development of exosomes as drug delivery systems faces numerous challenges. First, there is still a lack of efficient targeting ligands to enable the specific delivery of exosomes to tumor tissues while avoiding unnecessary accumulation in normal tissues^122^. Second, the efficient loading and precise evaluation of therapeutic cargo remain significant challenges^123^. The drug-loading efficiency is heavily influenced by the physicochemical properties of the drug and the process conditions, while current evaluation techniques (such as fluorescence labeling) are prone to various interferences, raising concerns about their accuracy^124^.

The clinical application of exosomes is still in the early stages of exploration. Rigorous, large-scale, prospective clinical studies based on exosomes remain scarce, with existing evidence mainly derived from small sample retrospective analyses or early exploratory trials. In addition, whether as a drug carrier or a therapeutic tumor vaccine, the efficacy and long-term safety of exosome-based strategies still require systematic evaluation^125^. Therefore, conducting systematic and rigorous clinical studies is crucial for assessing the risks and benefits of exosome-based therapies and, ultimately, for safely and effectively integrating them into the standard cancer treatment paradigm.

## Conclusions

In recent years, increasing research has focused on the role of exosomes in cancer metastasis. The intricate bidirectional communication between exosomes and the tumor microenvironment has become a critical challenge in the treatment of cancer metastasis. The changes in the microenvironment dictate the heterogeneity of exosomes. Therefore, further research is essential to explore the regulatory mechanisms underlying the exosome-mediated cell crosstalk.

As discussed herein, exosomes are involved in shaping both local invasion and distant metastasis in the TME, influencing processes such as angiogenesis, immune suppression, and ECM remodeling. However, the changes at local and distant metastatic sites carry distinct implications. For example, at the primary tumor site, exosomes regulate ECM remodeling by degrading ECM components, enhancing tumor cell invasiveness. In contrast, exosomes at distant PMN sites promote ECM formation that supports cell adhesion, enabling circulating tumor cells to survive and proliferate. Whether exosomes regulate changes at different sites through similar signaling pathways requires further investigation. Additionally, the mechanisms behind exosome-mediated organotropism—where tumors metastasize from different primary sites to the same organ—remain to be fully understood.

A thorough investigation of exosomes' roles in tumor chemoresistance is essential for developing strategies to reverse cancer drug resistance. Exosomal drug efflux pumps may be targeted in anti-resistance therapy. Exosomes also show potential in cancer immunotherapy as effective vaccines and targeted drug carriers. Additionally, their application in early cancer detection could lead to higher survival rates. Nevertheless, the clinical application of exosomes still faces several common challenges: (1) The high heterogeneity of exosomes derived from different tissues; (2) The absence of standardized protocols for the isolation and detection of exosomes; (3) The insufficiency of rigorously designed, large-scale clinical trials for their validation.

## Figures and Tables

**Table 1:** Changes in exosomal cargo and function induced by the hypoxic and acidic microenvironment.

TME stimulus	Cancer type	Altered cargo	Mechanism	Effect	Ref
Hypoxia	Breast cancer	lnc-MTRNR2L12-3	Src/FAK	Angiogenesis	126
Hypoxia	Breast cancer	miR-143-3p	RICTOR	Migration, invasion	127
Hypoxia	Breast cancer	lncRNA H19	DNMT1/ miR-497	Drug resistance	128
Hypoxia	Breast cancer	circSTAT3	miR-671-5p / NOTCH1	Drug resistance	129
Hypoxia	Breast cancer	lncRNA MIR210HG	PI3K/Akt/mTOR	Angiogenesis, invasiveness	130
Hypoxia	Breast cancer	miR-210-3p	NFIX-Wnt/β-catenin	Migration, invasion	131
Hypoxia	Pancreatic cancer	LARS1	HIF-1α/LARS1/mTOR	Angiogenesis	132
Hypoxia	Gastric cancer	LAMB2	ROCK1/CAV1/Rab11	Metastasis	133
Hypoxia	Colorectal cancer	miR-4299	HIF-1α/miR-4299/ZBTB4	Proliferation, metastasis	134
Hypoxia	Hepatocellular carcinoma	circHIF1A	HuR/PD-L1	Invasion, immune escape	57
Hypoxia	Glioma	miR-25-3p	PHLPP2/PI3K-AKT/mTOR	Immune escape	135
Hypoxia	Glioma	PKM2	ROS	Drug resistance	136
Hypoxia	Lung cancer	circPLEKHM1	PABPC1-eIF4G/OSMR	Immune escape	137
Hypoxia	Lung cancer	ALS2	Rab5/β-catenin	Angiogenesis	138
Hypoxia	Esophageal squamous cell carcinoma	circNRIP1	TRMT6	Migration, invasion	139
Hypoxia	Prostate cancer	miR-500a-3p	miR-500a-3p/FBXW7/HSF1	Metastasis	140
Acidosis	Melanoma	miR-214	↓VE-cadherin	Migration	23
Acidosis	Melanoma	miR-155, miR-210	glycolysis	Metastasis	141
Acidosis	Melanoma	CFL, GSN, HYOU1	\	Metastasis	142
Acidosis	Melanoma	miR-155-5p	SOCS1/JAK2/STAT3	Angiogenesis	143
Acidosis	Melanoma	\	↓Cisplatin activation	Drug resistance	144
Acidosis	Prostate cancer	Carbonic anhydrase IX	\	Metastasis	145
Acidosis	Hepatocellular carcinoma	miR-21, miR-10b	HIF-1α, HIF-2α	Proliferation, metastasis	146

**Table 2:** Integrin-mediated organ-specific metastasis.

Exosomal integrin	PMNSite	Target	Primary Tumor	Mechanism	Ref.
Integrin α6β4	Lung	HUVECs	Colorectal cancer	Facilitates proliferation and tubulogenesis	68
Integrin α6β4	Lung	Epithelial cells, fibroblasts	Breast cancer	Activates the expression of S100	67
Integrin α6β1	Lung	Fibroblasts	Breast cancer	Activates the expression of S100	67
Integrin αvβ3	Lung	\	Melanoma	Enhances tumor cell invasion	147
Integrin α2β1	Lung	Fibroblasts	Salivary adenoid cystic carcinoma	Induces the expression of p-Smad3 and activate the TGF-β signaling pathway	86
Integrin αvβ5	Liver	\	Colorectal cancer	Activates αvβ5/FAK/NF-κB signaling	148
Integrin αvβ5	Liver	Macrophages	Breast cancer	Promote metastatic niche formation	149
Integrin β4	Liver	Macrophages	Breast cancer	Enhances tumor cell invasion	149
Integrin α5	Bone	Osteoblasts	Breast cancer	Facilitates RUNX2 high-expressing breast cancer cell colonization in bone	150
Integrin αvβ6	Bone	\	Prostate cancer	Initiate the osteolysis	151
Integrin β3	Brain	Endothelial cells	Breast cancer, melanoma	Facilitates metastatic niche formation	67
Integrin β4	\	Prostate cancer cells	Prostate cancer	Enhances cell adhesion, migration, and invasion	152
Integrin αvβ6	\	TGFβ	Prostate cancer	Induces migration and invasion	153
Integrin αvβ3	\	Lung	Non-small-cell lung cancer	Induces polarization of M2-like macrophages	154
Integrin α5β1	\	Peritoneal mesothelial cells	Colorectal cancer, Epithelial ovarian cancer	Enhances cell adhesion and migration	71, 155

**Table 3:** Exosomes as potential biomarkers for the diagnosis and prognosis in different types of cancers.

Biomarker	Types of cancers	Sources of samples	Clinical applications	Ref
circRNA-100338	Hepatocellular carcinoma (HCC)	serum	risk indicator of pulmonary metastasis and poor survival	156
lncRNA CASC9	Hepatocellular carcinoma (HCC)	serum	early diagnosis	157
miRNA-720	Hepatocellular carcinoma (HCC)	serum	diagnoses small HCC and evaluate advance	103
miR-1275	Hepatocellular carcinoma (HCC)	serum	early diagnosis and prognosis	158
RAB22A	Multiple myeloma (MM)	serum	evaluates progression and relapse of MM	159
tsRNA tRF-3004a	Colorectal cancer (CRC)	serum	associated with metastasis, CEA levels, and nerve/vascular invasion	160
lncRNA DLEU1	Cervical cancer (CC)	serum	diagnoses, monitors recurrence and metastasis, and evaluates prognosis	161
miR-9-5p	Osteosarcoma (OS)	serum	early diagnosis	106
miR-223-5p	Pancreatic ductal adenocarcinoma (PDAC)	serum	diagnoses PDAC patients with sarcopenia	162
enolase 1	Breast cancer (BC)	serum	early diagnosis	163
syndecan-2	Breast cancer (BC)	serum	predicts chemotherapy response in obese BC patients.	164
RAB21	Follicular thyroid carcinoma (FTC)	serum	early diagnosis	165
Protein tyrosine phosphatase receptor-type O	Lung adenocarcinoma (LUAD)	saliva	assesses the risk of adverse prognosis for early LUAD	166
miRNA-1307-5p	Oral squamous cell carcinoma (OSCC)	saliva	predicts disease progression and prognosis	167
circPRMT5	Head and neck squamous cell carcinoma (HNSCC)	saliva	early diagnosis	168

**Table 4:** Exosome-mediated drug delivery systems

Sources of exosomes	Types of cancers	Therapeutic payloads	Effects	Ref
Cancer cell	Colorectal cancer	miRNA206	Target T cell immune receptor	169
Cancer cell	Breast cancer	5-FU	Induced apoptosis	170
Cancer cell	Breast cancer	3,3′-diindolylmethane, doxorubicin	Inhibit cancer stem cells-driven EMT	171
Cancer cell	Breast cancer	LINC02544 siRNA	Inhibit proliferation and metastasis	172
Cancer cell	Diffuse large B-cell lymphoma	BTK siRNA	Accelerate T cells activation	173
Mesenchymal stem cell	Glioblastoma	Rapamycin	Modulate blood-brain barrier penetration and VEGF axis	174
Mesenchymal stem cell	Pancreatic cancer	Gal9 siRNA, DOGEM, ICG	Chemo-photo-immunotherapy	111
Mesenchymal stem cell	Colorectal cancer	Anti-miR-146b Antisense oligonucleotide	Suppress Smad signaling and EMT	175
Adipose-derived stem cell	Pancreatic cancer	PD-L1 siRNA	Bind to CD133-positive pancreatic cancer cells and suppress PD-L1 expression	176
Adipose-derived stem cell	Colon cancer	PIN1 siRNA	Target sAPRIL and inhibit tumor growth and EMT	177
Serum	Glioblastoma	TanIIA, glycyrrhizic acid	Induce apoptosis	178
Serum	Colon cancer	PD-1	Significant tumor growth retardation and immune responses	179
Mesothelial cell	Gastric cancer	miR-338-3p	Inhibit the proliferation and adhesion	109
M1 macrophage	Breast cancer	REV, SR780Fe	Activate photodynamic therapy, ferroptosis	180
HEK293T cells	Colorectal cancer	KRAS siRNA, 3-BP	Restored chemosensitivity	112

## Abbreviations

TME: tumor microenvironment
TAMs: tumor-associated macrophages
CTCs: cancer circulating tumor cells
EE: early endosomes
LE: late endosomes
MVBs: multivesicular bodies
ILVs: multiple intraluminal vesicles
ESCRT: endosomal sorting complexes required for transport
EMT: epithelial-mesenchymal transition
CRC: colorectal cancer
ECM: extracellular matrix
MMPs: matrix metalloproteinases
NFs: normal fibroblasts
ICIs: immune checkpoint inhibitors
NK: natural killer
PMN: pre-metastatic niches
IMCs: immature myeloid cells
HIFs: Hypoxia-inducible factors
HCC: hepatocellular carcinoma
HNSCC: neck squamous cell carcinoma
CSCs: Cancer stem cells
CAFs: tumor-associated fibroblasts
MDSCs: myeloid-derived suppressor cells
MSCs: Mesenchymal stem cells
BMDCs: bone marrow-derived cells
DCs: dendritic cells
IL4R: nterleukin-4 receptor
TNBC: triple-negative breast cancer
PTX: paclitaxel
DFX: Deferasirox
BBB: blood-brain barrier
IPSCs: induced pluripotent stem cells
CTX: cetuximab
Tregs: regulatory T cells
3BP: 3-bromopyruvate
DNMT3A: DNA methyltransferase 3α
